# Enhancing Tribological Performance of Self-Lubricating Composite via Hybrid 3D Printing and In Situ Spraying

**DOI:** 10.3390/ma17112601

**Published:** 2024-05-28

**Authors:** Alessandro M. Ralls, Zachary Monette, Ashish K. Kasar, Pradeep L. Menezes

**Affiliations:** Department of Mechanical Engineering, University of Nevada Reno, Reno, NV 89557, USA; alessandroralls@gmail.com (A.M.R.); zmonette@gmail.com (Z.M.); akasar@nevada.unr.edu (A.K.K.)

**Keywords:** ABS, hBN, friction, wear, composites, additive manufacturing, fused deposition modeling

## Abstract

In this work, a self-lubricating composite was manufactured using a novel hybrid 3D printing/in situ spraying process that involved the printing of an acrylonitrile butadiene styrene (ABS) matrix using fused deposition modeling (FDM), along with the in situ spraying of alumina (Al_2_O_3_) and hexagonal boron nitride (hBN) reinforcements during 3D printing. The results revealed that the addition of the reinforcement induced an extensive formation of micropores throughout the ABS structure. Under tensile-loading conditions, the mechanical strength and cohesive interlayer bonding of the composites were diminished due to the presence of these micropores. However, under tribological conditions, the presence of the Al_2_O_3_ and hBN reinforcement improved the frictional resistance of ABS in extreme loading conditions. This improvement in frictional resistance was attributed to the ability of the Al_2_O_3_ reinforcement to support the external tribo-load and the shearing-like ability of hBN reinforcement during sliding. Collectively, this work provides novel insights into the possibility of designing tribologically robust ABS components through the addition of in situ-sprayed ceramic and solid-lubricant reinforcements.

## 1. Introduction

Since the inception of 3D printing, a variety of industries (e.g., automotive, aerospace, healthcare, and entertainment [1,2,3,4,5,6,7,8,9,10]) have benefitted from its rapid prototyping-like features. In its most basic sense, 3D printing can be thought of as a form of additive manufacturing (AM), which consists of fabricating whole components using a layer-by-layer additive approach [11]. The advantage of using 3D printing pertains to its ability to fabricate robust components whilst having little to no waste [12]. To add to the appeal of 3D-printed components, the material from failed/end-of-life parts can also be re-used multiple times (depending on the material), which helps promote cost-effectiveness and a circular economy [13,14]. Because of these benefits, there has been significant growth in the 3D printing field [15,16].

Out of the existing 3D printing methods, fused deposition modeling (FDM) is one technique that has arguably seen the greatest amount of industrial usage [17,18,19,20]. Also known as fused filament fabrication (FFF), the FDM process involves the controlled heating and extrusion of plastic filaments through a small nozzle to create a physical part [21]. With the plastic leaves through the nozzle, it is in a molten-like state beyond the material’s glass transition temperature [22]. At the start of the FDM process, molten plastic is placed onto a heated bed as the base material. After solidification, a second layer of molten plastic is extruded and added above/to the side of the first layer. By following the pre-set g-code program, the process continues until the final part is complete [17].

In the FDM process, thermoplastic-based materials are traditionally used. Out of the existing array of thermoplastic materials, acrylonitrile butadiene styrene (ABS) is one material that attracts a large amount of industrial and research attention [23,24]. This attraction is largely due to its unique structure, as it is lightweight, easy to manufacture, and has recyclable characteristics. In frictional applications, they can act as low-cost self-lubricating surfaces due to their low-shear strength [25,26]. ABS also has impressive mechanical properties due to its long chain connections [27]. Because of these features, FDM’d ABS components see usage in automotive parts, gears, mechanical links, and industrial screws [22,28,29].

In the existing literature, there is a variety of work that has focused on optimizing the mechanical and tribological properties of FDM’d ABS materials [23,28,30,31,32,33,34,35,36,37]. In most cases, the processing parameters (e.g., filament thickness, infill density, infill pattern, infill temperature, scanning speed, orientation of deposition, and infill angle [38]) tend to be varied, which can allow for more structurally robust components. However, despite the vast advancements made in process optimization, the viscoelastic nature of polymer components (unlike traditional metal and ceramic materials) results in their rapid degradation in mechanical-based applications [39].

**Figure 1:**
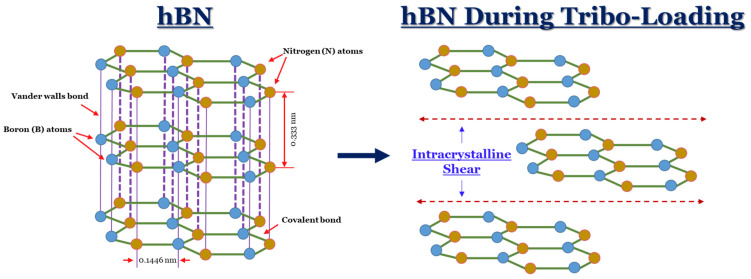
A visual depiction of the atomic structure of hBN alongside its shearing mechanism during rubbing contacts adapted from the work of Dadvand and Savadogo [56].

One method to improve their performance is adding reinforcement materials to the ABS matrix. In the literature, this is typically performed by mixing the reinforcement with the ABS filament before printing [40,41,42,43,44]. Although the concept of reinforcement addition is a well-known subject (especially for metallic-based materials [45,46]), they typically serve the role of either matrix stiffeners or solid lubricants [25]. In the case of matrix reinforcements, hard materials (which are typically either metallic-based or ceramic-based, such as alumina (Al_2_O_3_) [47]) serve the role of promoting a matrix-stiffening-like effect [25,41]. The addition of such reinforcements can be advantageous in contact/mechanical-based applications, which can help support the distribution of external loads and reduce material loss. These findings have been reported in FDM-related studies, such as in the work of Singh et al. [48], who used Al_2_O_3_ and SiC as reinforcements for ABS-FDM parts. On the other hand, lamellar/easily shearable reinforcement materials can provide lubricating-like effects in contact-based applications [49]. An example of this approach can be seen in the work of Keshavamurthy et al. [50]. In their work, the tribological response of FDM-printed ABS-containing graphite was investigated. Before deposition, the graphite powder was blended with the ABS pellets and extruded as a single filament. Their findings indicated that the layer-like structure of graphene allowed for a lubrication-like effect, which improved tribological performance. Aside from graphite, other materials, such as hexagonal boron nitride (hBN), are also investigated due to their similar lamellar-like crystal structure [51,52]. In each layer, the boron (B) and nitrogen (N) molecules are covalently bonded. However, between each layer, weak van der Walls forces exist. In sliding applications, these weak van der Walls forces allow for the layers to easily shear, which can improve frictional resistance [53]. A visualization of this phenomenon can be seen in Figure 1. Although this is one material from the existing array of self-lubricating composites, they all fundamentally serve to improve frictional resistance, which ultimately can help enhance component longevity [54,55].

Based on this information, the purpose of this work is to understand the influence of Al_2_O_3_ and hBN on the tribological performance of FDM’d ABS. Evaluations of ABS’s structural features and mechanical properties will be made to provide supporting evidence for the tribological mechanisms that take place. Particularly, the frictional response can be assessed. To achieve this, this work focuses on the independent and simultaneous addition of Al_2_O_3_ and hBN to the ABS matrix through a novel in situ spraying technique that eliminates the need for matrix-to-filler mixing. The intention of adding Al_2_O_3_ and hBN is to understand the interactions of matrix reinforcement (hard and soft) to the FDM-printed composite. It is expected that Al_2_O_3_ provides a load-bearing type of effect, whereas hBN acts as a solid lubricant. When combined, the combination of these features reduces the influence of direct tribological loads, which, in turn, reduces the frictional response. In doing so, moving mechanical assembly components in applications such as textile drying machines, bushings, and gears, which oftentimes cannot use external lubricants, can benefit due to the self-replenishable and frictional-reducing characteristics of the proposed FDM components [25,39]. Through this work, key insights into the role of these in situ-sprayed composites can be determined, which can help to further the scientific advancement of the FDM field.

## 2. Experimental Details

### 2.1. Fused Deposition Modeling

**Figure 2:**
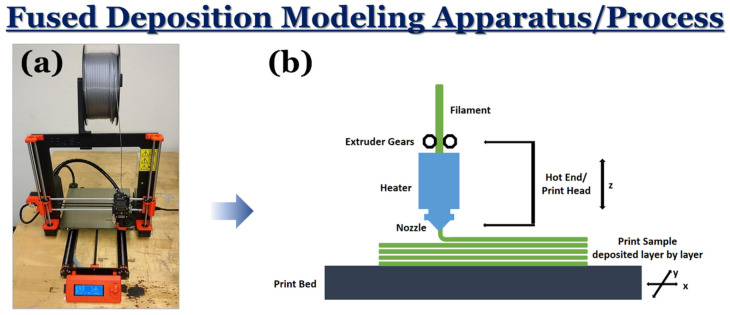
A schematic depicting the (**a**) FDM 3D printing equipment and (**b**) printing process performed in this work.

All samples in this study were printed using a Prusa I3 Mk3 3D printer (Prusa Research, Prague, Czech Republic) with a 0.4 mm diameter nozzle (Figure 2). To create the design of the samples, a computer-aided design (CAD) model was prepared using SolidWorks software (SolidWorks Version 13000, Waltham, MA, USA). For each sample, the dimensions were set to 30 mm × 30 mm × 8 mm. After being designed, the model was then sliced using the Slic3r (Version 1.3.0, Prusa Edition) slicing program.

For all experiments, the base ABS material consisted of an ABS Pro Series filament 1.75 mm diameter by Matter Hackers (Matter Hackers Foothill Ranch, Lake Forest, CA, USA). During the fabrication process, the layer height was set to 0.25 mm while the nozzle temperature was set to 255 °C. To ensure proper heat distribution, the bed temperature, which was placed below a removable stainless steel polyetherimide (PEI) sheet, was set at 100 °C. This sheet served two different functions. First, it prevented the composite material from making contact with the bed. Aside from this function, the PEI sheet also allowed for the printed component to be easily removed [57]. For a more detailed view, the printing parameters used are listed in Table 1. During printing, the samples were printed using an internal grid infill pattern and a top/bottom rectilinear infill pattern. For reference, the FDM machine was set to achieve a 100% infill density.

**Figure 3:**
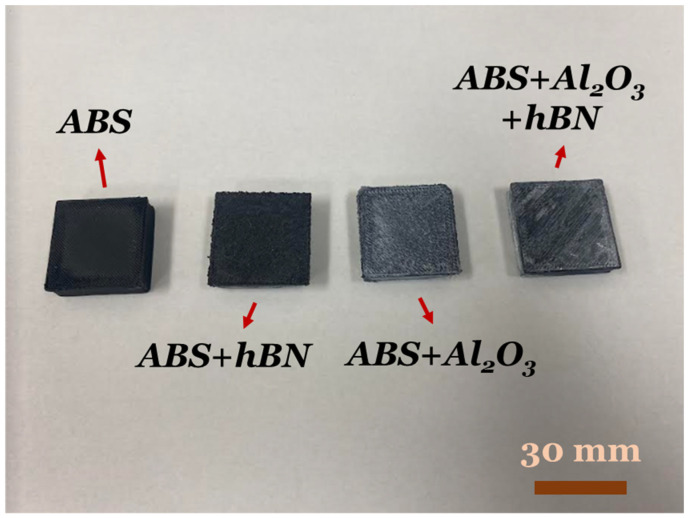
A visual representation of the ABS, ABS + Al_2_O_3_, ABS + hBN, and ABS + hBN + Al_2_O_3_ specimens used in this work.

To fabricate the ABS composites, a novel in situ spraying method was used. This method involves spraying suspended material in isopropyl alcohol while printing. The advantage of this method lies in the fact that material additives can be added between every layer instead of just being integrated into the filament. Overall, four sets of samples were printed—one for each additive sample and one as the base sample with no additive—as mentioned in Table 2. The additives used consisted of Al_2_O_3_ (US Nano Research, Houston, TX, USA) and hBN (M K Impex Corp., Mississauga, ON, Canada), with average particle sizes of 500 nm and 70 nm. From this point, moving forward, the samples are referred to as ABS, ABS + Al_2_O_3_, ABS + hBN, and ABS + hBN + Al_2_O_3_, as indicated in Table 2. In the spray bottle, 2 g of the additive was mixed with 130 mL of isopropyl alcohol. The spray bottle was then agitated by hand to ensure that the solution was dispersed throughout the ethanol solution. After agitation, the mixture was sprayed on the top of the print surface through custom stops in the g-code program. To prevent the freshly deposited filament from being distorted/warped from the ethanol-containing reinforcement solution, the solution was sprayed 10 s after the layer was deposited. It should be mentioned that during spraying, factors such as potentially varied applied forces to the spray bottle trigger, the varied flow of the particles at the moment during spraying, and the variation in ethanol in the applied spray could affect both the weight and uniformity of the reinforcements sprayed between each layer. This presents a potential limitation in this new technology. Nonetheless, to minimize these potential byproducts, the spray bottle was agitated before each intralayer spray to prevent particle sedimentation. Furthermore, the misted spray from the aerosolized spray bottle was performed approximately 8 inches away from the printed layer. This was performed to maximize the chance that the reinforcements were uniformly sprayed onto each layer. After being sprayed, the alcohol in the additive mixture evaporated almost immediately due to the residual heat of the printed layer. Despite the 10 s intralayer layer spray dwell time, such interactions could have a detrimental effect on the print quality due to altered cooling rates. One potential approach to decouple the influence of temperature during the spray was to use a gaseous substance as the transportation media for the reinforcements instead of ethanol. Although this approach was outside the scope of this work, this approach can theoretically mitigate the cooling effects that the ethanol gas causes during spraying. Nonetheless, after the layer was deposited, a subsequent layer was then printed directly on top of the sprayed composite. This process continued until this part was completed. Afterward, the printer was turned off, and the print was allowed to cool directly on the printer bed. To better visualize the finalized parts, photographs of the ABS, ABS + Al_2_O_3_, ABS + hBN, and ABS + hBN + Al_2_O_3_ are shown in Figure 3, indicating that high printing quality was achieved. Post-printing, the surfaces of the samples were then characterized using an Axioscope Fluorescence Microscope (Zeiss, White Plains, NY, USA) and a JSM-6010LA InTouchScope Scanning Electron Microscope (SEM) coupled with Energy Dispersive Spectroscopy (EDS) capabilities (JOEL, Tokyo, Japan).

### 2.2. Mechanical Testing

**Figure 4:**
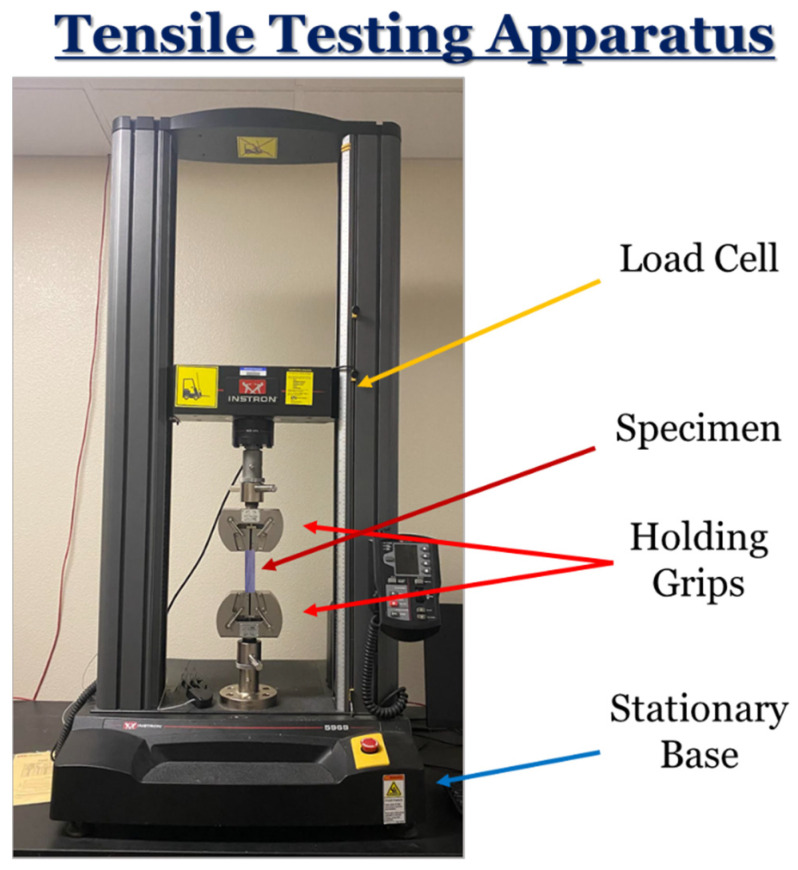
The mechanical testing apparatus used to perform tensile-loading tests.

Tensile tests were completed to determine the mean ultimate tensile stress (UTS) of the four sample sets. The intention of performing these tests was to provide additional support for the tribological mechanisms in this work. In particular, the quality of interparticle bonding can be quantifiably obtained, which can provide useful insights and correlations to the tribological findings. However, in pure mechanical applications, Al_2_O_3_ and/or hBN reinforcements are added. All tension tests were performed on an Instron Model 3366 Dual Column Uniaxial Material Testing Machine (Instron, Norwood, MA, USA) (Figure 4) with 0.057 μm displacement precision, 0.001 N force accuracy, and 10 kN load force capacity. All samples were secured in the jaws of mechanical wedge action grips, and tension tests were run at a strain rate of 6.5 × 10^−4^ s^−1^ with an associated grip speed of 4.46 mm/min. The testing environment was set to room temperature (~25 °C). Each tension sample was then pulled until fracturing occurred. To ensure repeatability, three printed dog bones of each specimen were tested and averaged, with the standard deviation scale bar representing the findings from all three tests. These samples were designed as per the ASTM D638 standard. The results from these tests were used to determine the mean ultimate stress, mean yield stress, and effective modulus of elasticity along the longitudinal loading direction. Furthermore, the modulus of toughness was also determined in this analysis by integrating the area beneath the stress–strain curve.

### 2.3. Tribological Experimentation

**Figure 5:**
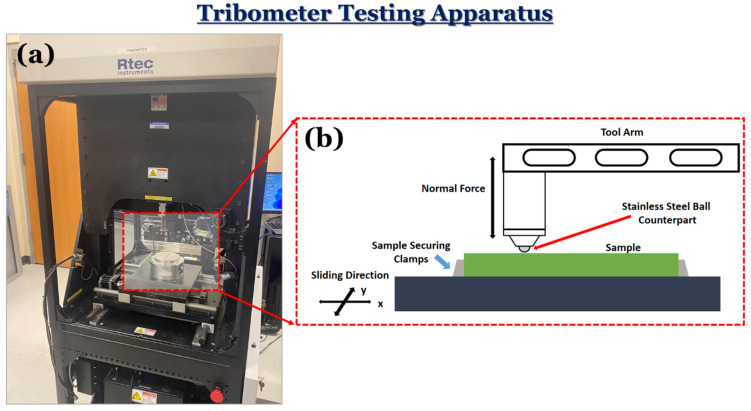
A schematic of the (**a**) tribometer equipment and (**b**) tribological testing procedure used in this work.

The dry sliding tests were performed using a Rtec Multifunction Tribometer 5000 (Rtec Instruments, San Jose, CA, USA). The tribometer features a high-resolution capacitance load sensor, which allows for the precise calculation of the coefficient of friction (COF) during sliding. Furthermore, the counterpart displacement in the z-axis was monitored. The samples were subjected to a reciprocating ball on a flat sliding test against a 6.35 mm diameter stainless steel ball as a counterpart. A schematic of the experimental setup can be seen in Figure 5. The reciprocating tests were conducted for 50 cycles, with the wear track being set to 10 mm. The tribological tests were conducted at three different normal loads of 10 N, 50 N, and 100 N with a corresponding sliding velocity of 0.5 mm/s. Similar to the approach mentioned in Section 2.2, each specific experiment was tested and averaged at least three times on different regions of the same sample for repeatability. For reference, the purpose of this work is to solely evaluate the change in friction from the composite additives.

## 3. Results and Discussion

### 3.1. Surface Features

**Figure 6:**
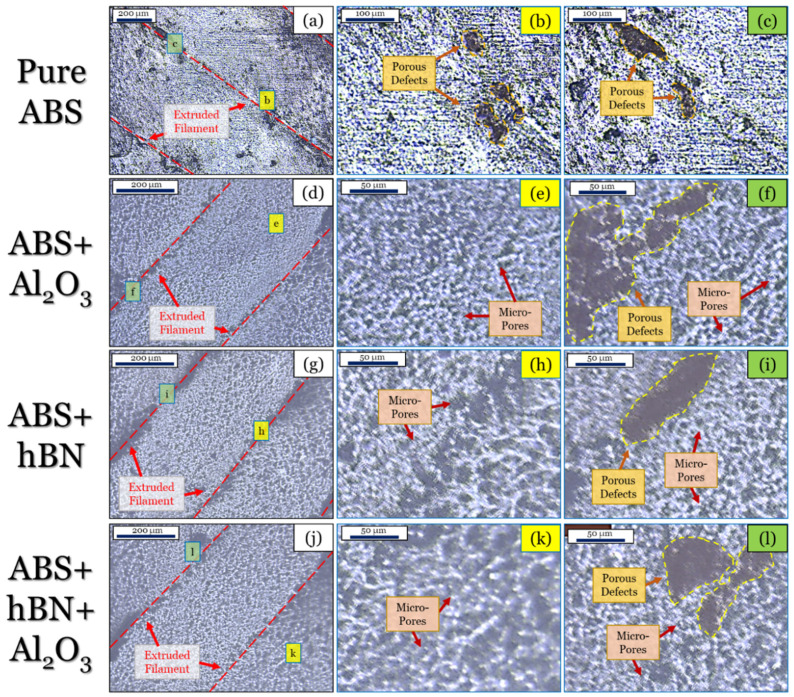
Optical micrographs of the (**a**–**c**) ABS, (**d**–**f**) ABS + Al_2_O_3_, (**g**–**i**) ABS + hBN, and (**j**–**l**) ABS + hBN + Al_2_O_3_ surfaces.

The surface features of the pure ABS and ABS composite parts obtained from optical imaging are shown in Figure 6. In this figure, a macroscopic view of the topmost surface, alongside various zoomed magnifications of different regions along the surface, is shown. These magnified sections correspond to the green and yellow boxes shown in Figure 6a,d,g,j. For reference, the macroscopic images of the ABS, ABS + Al_2_O_3_, ABS + hBN, and ABS + hBN + Al_2_O_3_ surfaces are shown in Figure 6a,d,g,j. The magnified regions of the ABS, ABS + Al_2_O_3_, ABS + hBN, and ABS + hBN + Al_2_O_3_ macroscope figures are shown in Figure 6b,c,e,f,h,i,k,l. For the pure ABS part (Figure 6a), a typical line-by-line arrangement of the ABS layer can be seen [58]. Within the deposited layer, small pores can be seen, which are likely attributed to the nozzle temperature during deposition. Since the glass transition point of ABS is ~112 °C [59], the 225 °C processing temperature could have affected the viscosity of the filament [60,61]. As a consequence, rapid cooling could promote an uneven dissipation of heat, which could produce porous defects. Such defects are typical for any thermal-based AM process. Inspecting between the deposited layers (Figure 6b,c), it can be seen that more porous defects are present. Since the printed material is produced layer-by-layer, air gaps can form in between each deposited filament [62]. Once Al_2_O_3_ is added (Figure 6d–f), it can be seen that the surface features drastically change. From a macroscopic view (Figure 6d), it is evident that there is a larger quantity of pores within the filament. Although relatively smaller in size (compared to the pure ABS surface), the formation of these pores can be attributed to the interactions of the Al_2_O_3_ reinforcement on the ABS layer during the cooling process. Likely, the high-temperature stability of Al_2_O_3_ (with a melting point of ~2072 °C [63]) altered the cooling rates of the surrounding layer. Similarly, the interactions of the alcohol with the heat dissipation of the layer could have also resulted in these findings. Along the edges of the extruded layer, large pores can also be seen. Appearing much larger in size compared to the pure ABS sample, it is likely that the interactions of the Al_2_O_3_ reinforcement and alcohol resulted in large amounts of trapped air during the deposition process. The ABS + hBN (Figure 6g–i) and ABS + Al_2_O_3_ + hBN (Figure 6j–l) samples also had similar surface features as the ABS + Al_2_O_3_ surface. However, for the ABS + hBN specimen, the size of the micropores appears to be slightly larger, with the contacting regions of the layer having larger voids. On the other hand, the ABS + hBN + Al_2_O_3_ specimen had micropores that were more closely resembling the ABS + Al_2_O_3_ surface. Collectively, these findings validate the fact that the reinforcement particles had some influence on the cooling kinetics of the ABS material.

**Figure 7:**
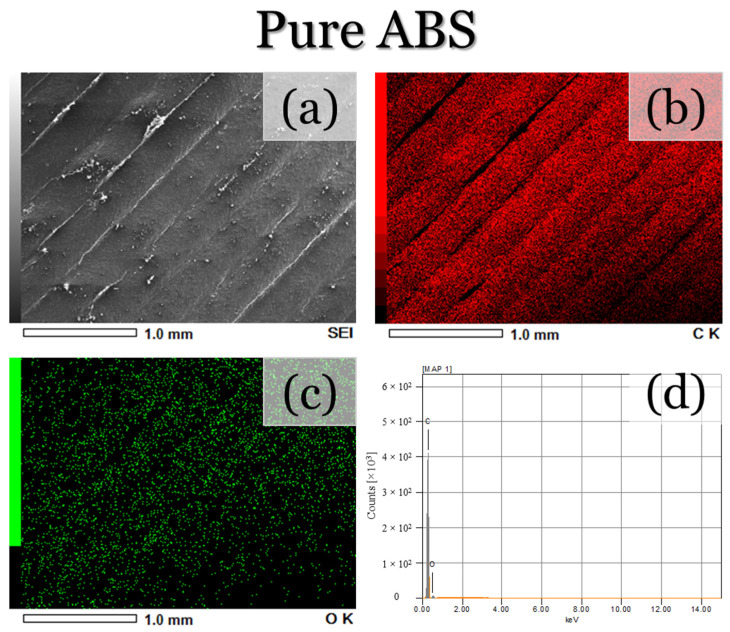
(**a**) SEM and (**b**–**d**) areal EDS map micrographs/EDS spectra of the ABS-printed surface.

**Figure 8:**
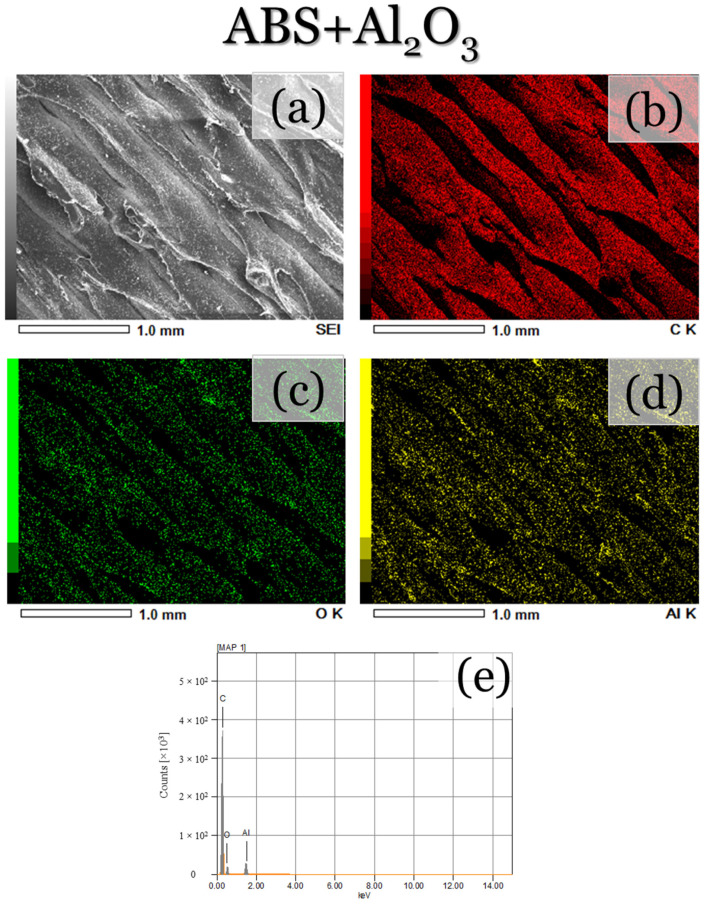
(**a**) SEM and (**b**–**e**) areal EDS map micrographs/EDS spectra of the ABS + Al_2_O_3_-printed surface.

**Figure 9:**
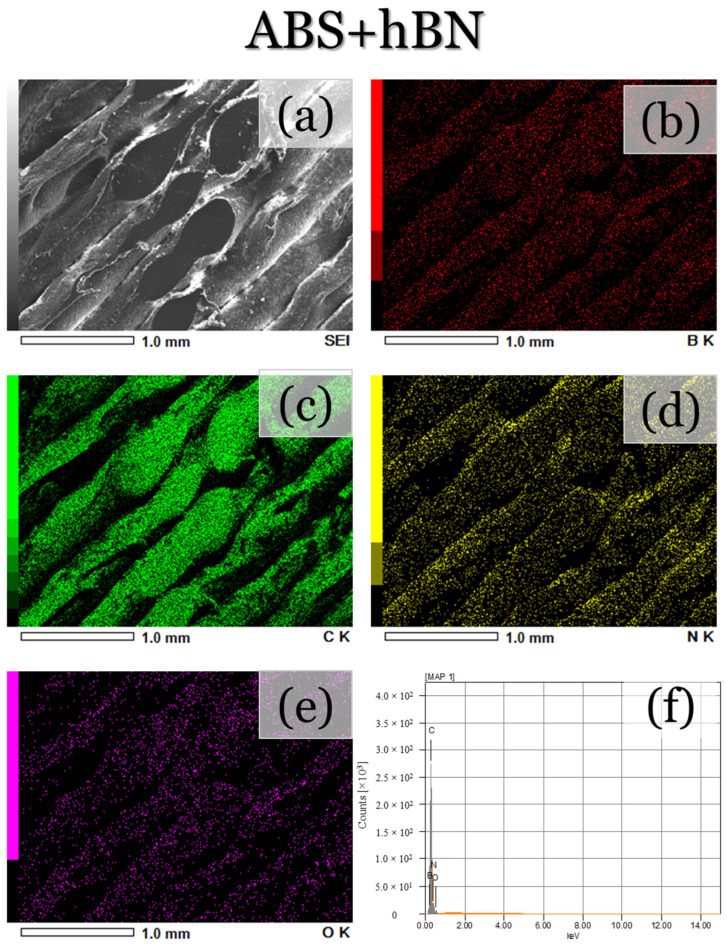
(**a**) SEM and (**b**–**f**) areal EDS map micrographs/EDS spectra of the ABS + hBN-printed surface.

**Figure 10:**
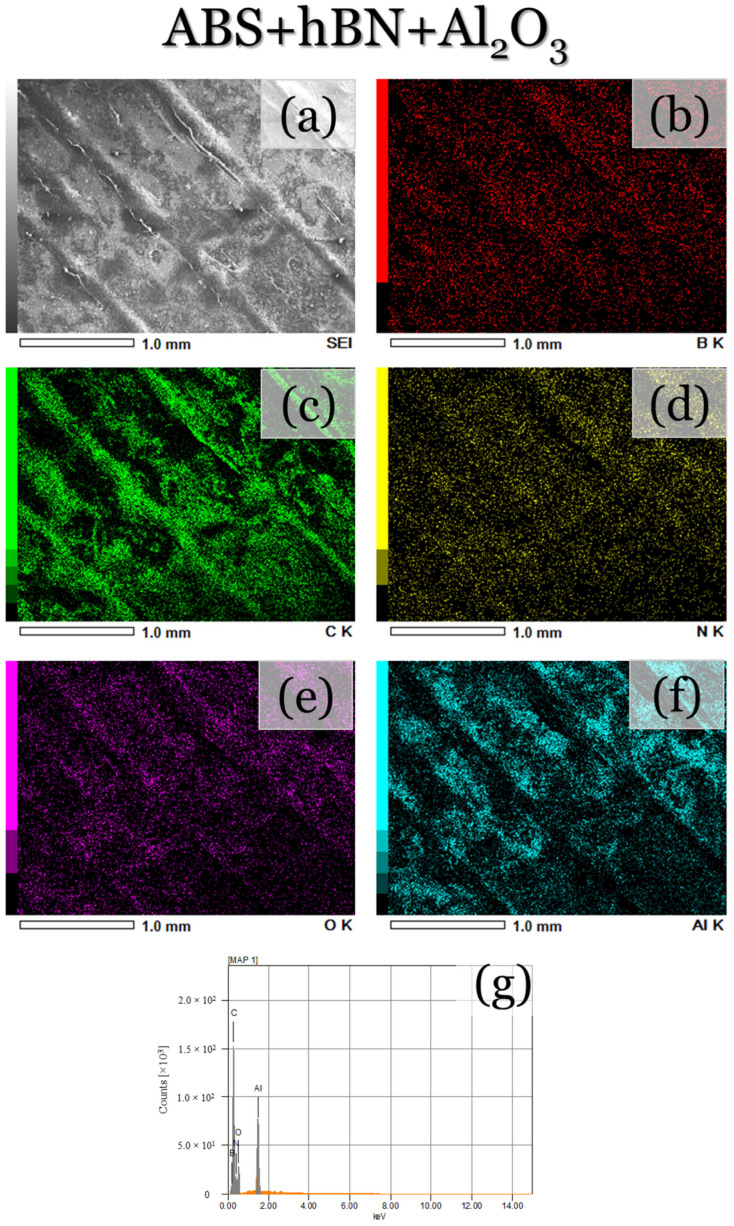
(**a**) SEM and (**b**–**g**) areal EDS map micrographs/EDS spectra of the ABS + hBN + Al_2_O_3_-printed surface.

To further validate the presence of the composite reinforcements, as well as their distribution along each printed layer, SEM micrographs and EDS map scans were performed along the surface of each printed specimen. For the ABS specimen (Figure 7a), the same topographical features, as shown in Figure 6a–c, can be seen. The EDS maps and spectra (Figure 7b–d) also depict the presence of carbon, which is to be expected due to the polymeric structure of ABS. Oxygen can also be seen, which is likely attributed to the rapid cooling and oxidation of the filament during deposition. On the other hand, the ABS + Al_2_O_3_ specimen (Figure 8a) depicts less smooth topographical features, which is likely due to the altered cooling rates from the ethanol. The EDS maps/spectra (Figure 8b–e) also show the presence of carbon, oxygen, and aluminum elements, which validate that Al_2_O_3_ was indeed deposited. For the aluminum element (Figure 8d), it appears that it is fairly uniformly dispersed, with only a few regions having a somewhat higher concentration of Al_2_O_3_. Since the spray process was performed manually, it can be expected that the complete uniform distribution of particle reinforcement would be difficult to achieve. Factors such as the varied applied forces to the spray bottle trigger, the flow of the particles at the moment of spraying, as well as the amount of ethanol in the applied spray, could have contributed to these results. Nonetheless, fairly uniform Al_2_O_3_ distribution was achieved. It should be mentioned that these factors can also make the determination of the sprayed particle quantity complex to assess. However, in this assessment, the criteria for deposition success depends on the amount of uniform particle dispersion along the ABS deposited layer. For the ABS + hBN (Figure 9) and ABS + hBN + Al_2_O_3_ (Figure 10) specimens, the presence of hBN and Al_2_O_3_ can be seen. It is interesting to note that the dispersion of hBN particles appears to be more evenly dispersed compared to the Al_2_O_3_ particles. Since the hBN particles are smaller in size, there naturally is a larger quantity of individual particles during spraying. Because of this increase in quantity, there is a greater likelihood that they will be evenly distributed. Nonetheless, these findings collectively show that intra-layer reinforcement spraying can result in a somewhat uniform deposition of particle reinforcements for FDM-printed ABS.

### 3.2. Mechanical Properties

**Figure 11:**
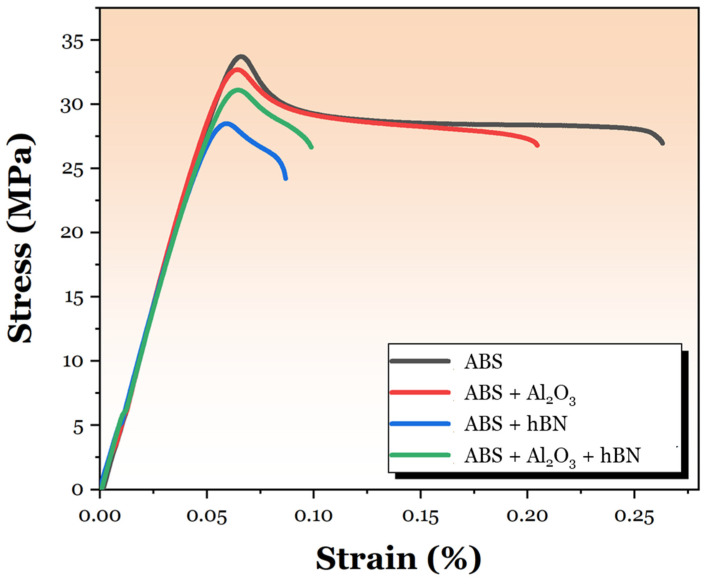
The tensile stress–strain plots of the ABS, ABS + Al_2_O_3_, ABS + hBN, and ABS + hBN + Al_2_O_3_ specimens.

Figure 11 shows the tensile stress–strain plots of the ABS, ABS + Al_2_O_3_, ABS + hBN, and ABS + Al_2_O_3_ + hBN materials. It can be seen that the ABS specimen had higher ductility and higher strength relative to the composite specimens. This finding can be likely attributed to the lower presence of micropores, as they reduce the likelihood of premature fracturing [64]. Once Al_2_O_3_ is added, the ductility and strength slightly decrease. Although Al_2_O_3_ should have a positive effect [65], this decrease can likely be attributed to the lack of cohesive bonding between the ABS layers due to the interactions of the Al_2_O_3_ composite and alcohol. Once hBN is added, the strength and ductility decrease again. According to Quill et al. [66], hBN exhibits little-to-no chemical bonding to ABS materials due to the difference in adhesion characteristics. Since the surface had a high amount of micropores (Figure 6g–i), the influence of hBN particles decreased the mechanical strength. When Al_2_O_3_ was added with the hBN reinforcement (i.e., ABS + Al_2_O_3_ + hBN), the strength and ductility slightly increased relative to the ABS + hBN sample. This increase suggests that by decreasing the hBN content from 2 g (in the ABS + hBN sample) to 1 g (in the ABS + Al_2_O_3_ + hBN sample), the strength and ductility of the ABS specimen can be improved.

**Figure 12:**
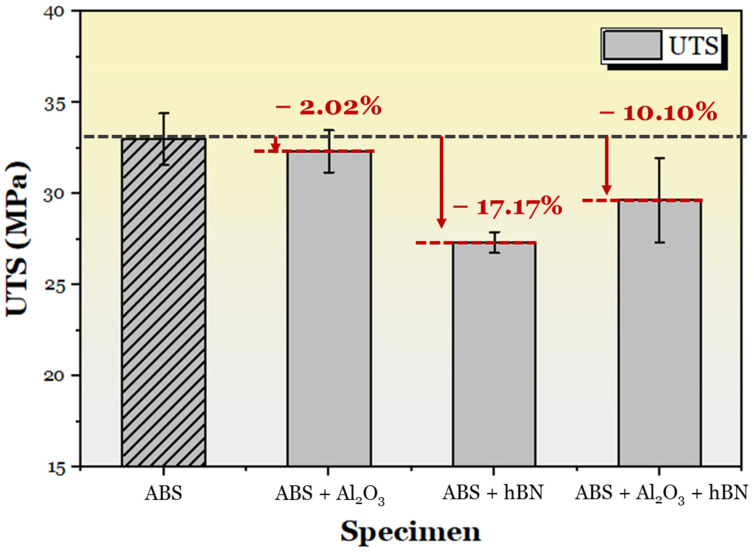
The change in UTS for the tensile-tested ABS, ABS + Al_2_O_3_, ABS + hBN, and ABS + hBN + Al_2_O_3_ specimens.

The ultimate tensile strength (UTS) from the tensile tests was also obtained, as shown in Figure 12. It can be seen that, across all composite specimens, the UTS decreased compared to the non-composite ABS specimen. In particular, for the ABS + Al_2_O_3_ specimen, there was a 2.02% decrease, whereas the ABS + hBN and ABS + Al_2_O_3_ + hBN specimens decreased by 17.17% and 10.10%.

**Figure 13:**
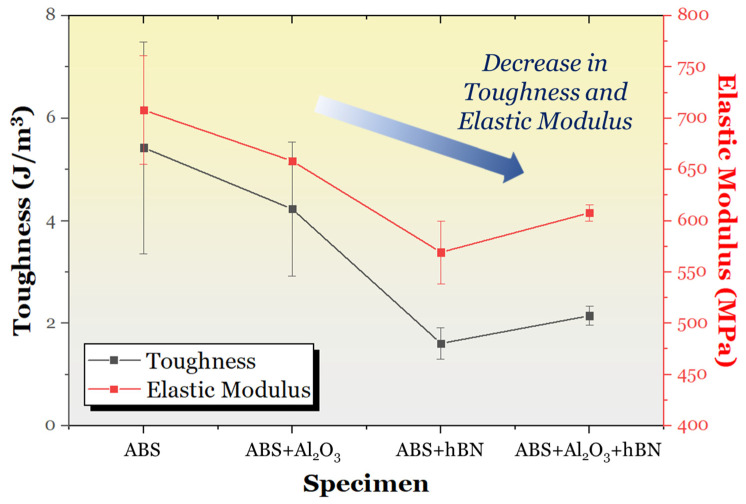
The evolution of toughness and elastic modulus for the ABS, ABS + Al_2_O_3_, ABS + hBN, and ABS + hBN + Al_2_O_3_ specimens.

Aside from assessing the UTS, the toughness and elastic modulus of the ABS and ABS composite specimens were obtained. These findings are shown in Figure 13. The ABS specimen had the greatest resistance to elastic deformation, followed by the ABS + Al_2_O_3_, ABS + Al_2_O_3_ + hBN, and ABS + hBN specimens. The toughness also followed a similar decreasing trend, which validated the visual analysis in Figure 11.

**Figure 14:**
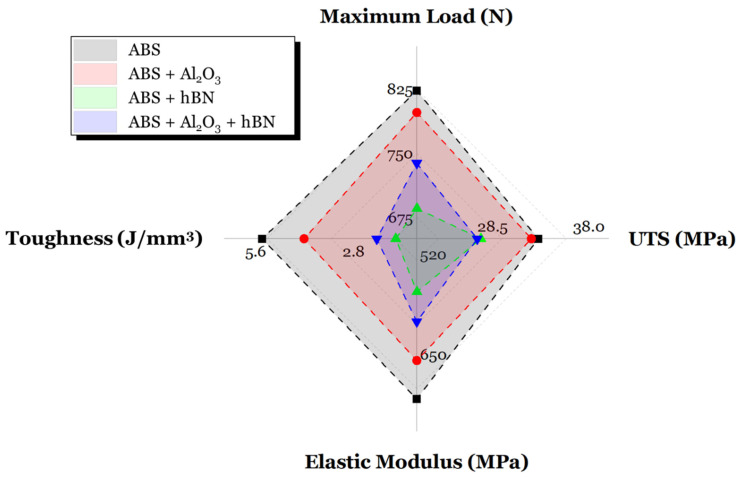
A comprehensive view of the relation between reinforcement addition to the layer and tensile-based mechanical properties.

A culmination in the mechanical characteristics of ABS with and without reinforcement additions is shown in Figure 14. More specifically, in this figure, the maximum load, toughness, UTS, and elastic modulus of all specimens are shown. The intention of creating such a figure is to understand the implications of reinforcement additions to the mechanical strength of 3D-printed ABS composites. It is clear to see that the area of the plot decreases once reinforcement is added, indicating a reduction in mechanical strength. Although Al_2_O_3_ does not have as much of a detrimental effect as hBN, the addition of these reinforcements between each ABS layer negatively affects the cohesion of each ABS layer. Overall, these findings indicate that adding an interlayer reinforcement is not a feasible method to improve ABS mechanical strength. Rather, the addition of interlayer reinforcement increases the likelihood of fracturing due to the loss of molecular orientation and the appearance of voids [67].

### 3.3. Tribological Performance

#### 3.3.1. ABS-Al_2_O_3_ Composites

**Figure 15:**
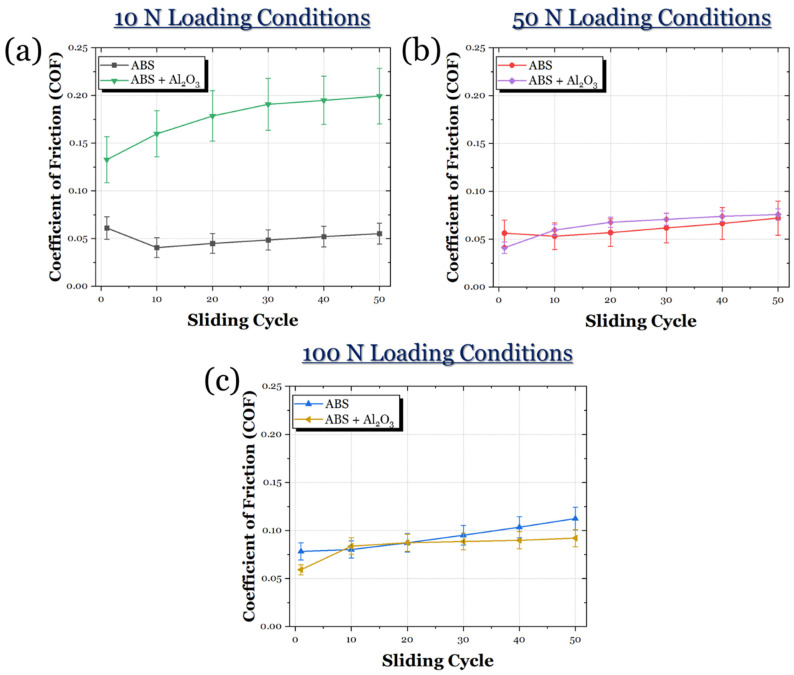
The frictional response of the ABS and ABS + Al_2_O_3_ specimens in (**a**) 10 N, (**b**) 50 N, and (**c**) 100 N loading conditions.

The evolution of friction for the ABS and ABS + Al_2_O_3_ specimens in 10 N, 50 N, and 100 N loading conditions can be seen in Figure 15. In the 10 N loading condition (Figure 15a), it can be seen that the COF for the ABS + Al_2_O_3_ specimen is much greater than the ABS specimen. In fact, throughout the sliding period, the COF gradually increases until it reaches a steady state. For the ABS specimen, the friction initially decreases, then remains largely in a steady state. However, in the 50 N loading condition (Figure 15b), the frictional curve of the ABS + Al_2_O_3_ greatly decreases to a value similar to the ABS specimen. However, it can be seen that the ABS specimen visually has a slightly lower frictional response. Once the load is increased to 100 N (Figure 15c), the ABS + Al_2_O_3_ specimen shows a reduced frictional response compared to the ABS specimen. It can be seen that the ABS specimen has a slight increase in friction throughout the sliding time, whereas, for the ABS + Al_2_O_3_ specimen, the frictional response is relatively constant.

**Figure 16:**
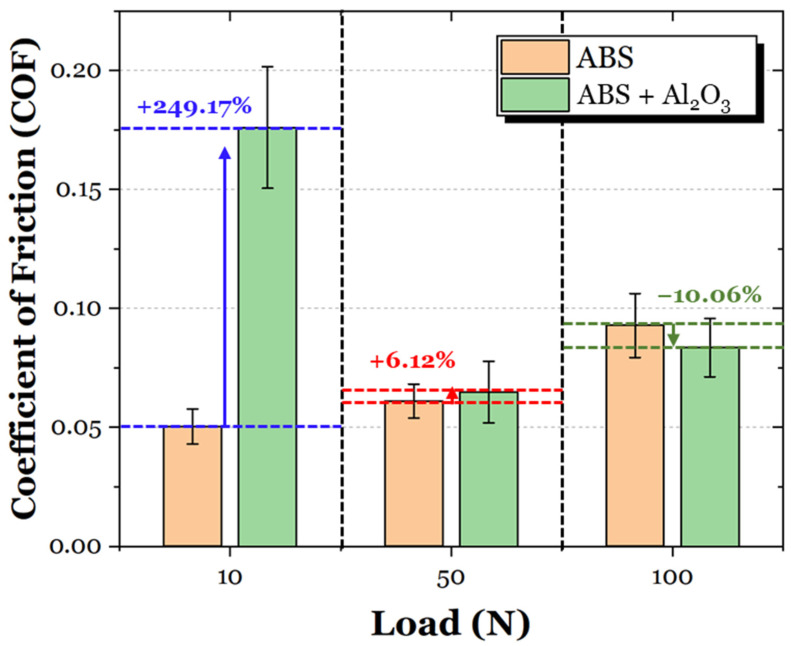
The average COF of the ABS and ABS + Al_2_O_3_ specimens in 10 N, 50 N, and 100 N loading conditions.

The average coefficient of friction (COF) values of the ABS and ABS + Al_2_O_3_ specimens are shown in Figure 16. For the 10 N loading condition, the ABS + Al_2_O_3_ specimen had a 249.17% increase in friction. However, as the load increased, the difference in COF also decreased. In particular, for the 50 N condition, the Al_2_O_3_ specimen had a 6.12% increase in friction, whereas for the 100 N condition, the friction decreased by 10.06%. It is interesting to note that unlike the ABS specimen, which has a gradual increase in friction as a function of load (which can be attributed to the increase in contact area during sliding), the COF of the ABS + Al_2_O_3_ specimen decreases, then slightly increases again. These findings suggest that Al_2_O_3_ has some influence on the frictional characteristics in varying loading conditions.

**Figure 17:**
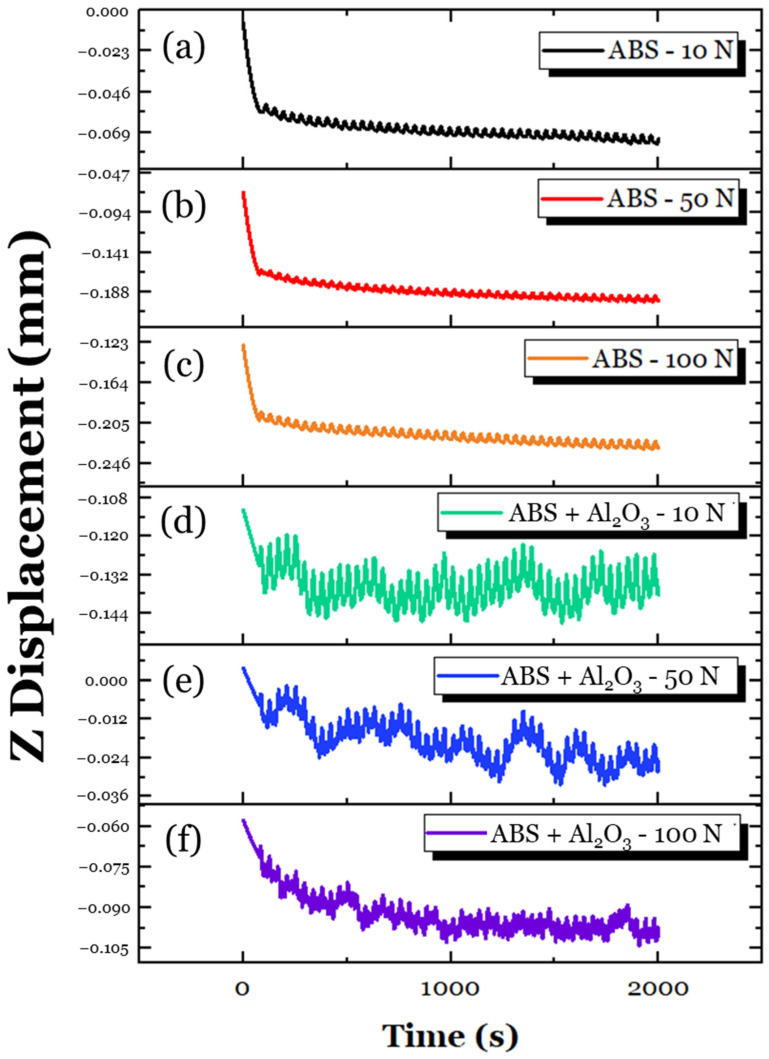
The variation in Z-displacement for the ABS specimen at (**a**) 10 N, (**b**) 50 N, and (**c**) 100 N loading conditions; the variation in Z-displacement for the ABS + Al_2_O_3_ specimen at (**d**) 10 N, (**e**) 50 N, and (**f**) 100 N loading conditions.

To obtain a clearer understanding of these interactions, the change in counterball vertical displacement (which is referred to as Z displacement) for all the tested samples during sliding was obtained, as shown in Figure 17. For the ABS specimen (Figure 17a–c), the Z displacement across all loads was fairly consistent. Since the tribological contacts of polymers tend to be mainly elastic [39], it can be insinuated that the sliding system was largely in a steady state. For the ABS + Al_2_O_3_ specimen (Figure 17d–f), there were greater fluctuations with the Z displacement compared to the ABS specimen. This was especially evident at the 10 N load (Figure 17d), which showed rapid oscillations compared to the ABS specimen (Figure 17a). Interestingly, as the load increased, the Z-displacement became more stable, with the 100 N load condition (Figure 17f) having the most stable Z-displacement. The initial variation can be attributed to the possible influence of third bodies along the surface. Since the ABS + Al_2_O_3_ specimen has a lessened elastic modulus/toughness, fragments of Al_2_O_3_ were likely formed, which altered the tribological contacts of the counter ball. This finding could explain the increase in friction. However, as the load increased, the fragmented Al_2_O_3_ reinforcement was likely adhesively re-embedded into the wear track. This, in turn, allowed for greater load-carrying support [68], which, in turn, reduced the COF.

**Figure 18:**
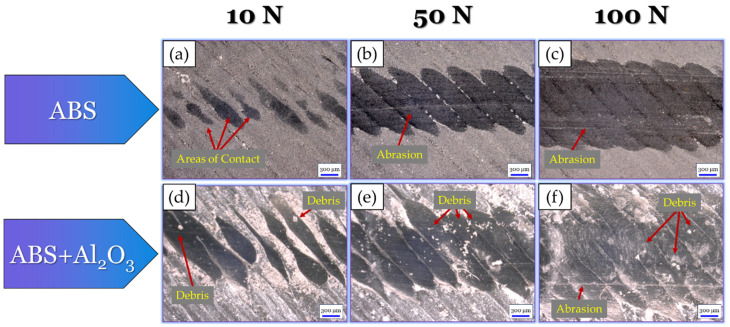
Optical micrographs of the (**a**–**c**) ABS and (**d**–**f**) ABS + Al_2_O_3_ wear tracks after tribo-sliding.

To further understand the tribological mechanisms that took place, optical micrographs of the wear tracks for each specimen were obtained, as shown in Figure 18. For the ABS specimen at the 10 N load (Figure 18a), there were limited areas of contact within the wear track. This finding can be attributed to the hill-like surface texture of the ABS print. At the 50 N (Figure 18b) load, the wear track became more uniform due to the greater area of contact from the tribo-load. Evidence of slight abrasion could also be seen in the central region of the wear track. At the 100 N load (Figure 18c), evidence of complete surface deformation could be seen. This finding could be due to the high loads from the tribo-test, which allowed for full contact despite the surface’s hill-like texture. Within the wear track, a greater number of parallel scratch lines could also be seen, which suggests that a greater amount of abrasive wear took place. For the ABS + Al_2_O_3_ specimen at the 10 N load (Figure 18d), the contours appear to be intact. However, they had a thinned-like appearance due to the influence of sliding. Small debris can also be seen within the wear track, validating the fact that debris had a role during tribo-sliding. At the 50 N load (Figure 18e), debris could still be seen within the wear track. However, the initial grooves from the surface texture were diminished, suggesting that more uniform tribo-contacts took place. At the 100 N load (Figure 18f), the wear track was largely smooth with slight signs of abrasion. Wear debris can also still be seen within the wear track, indicating that they had some role during sliding.

#### 3.3.2. ABS-hBN Composites

**Figure 19:**
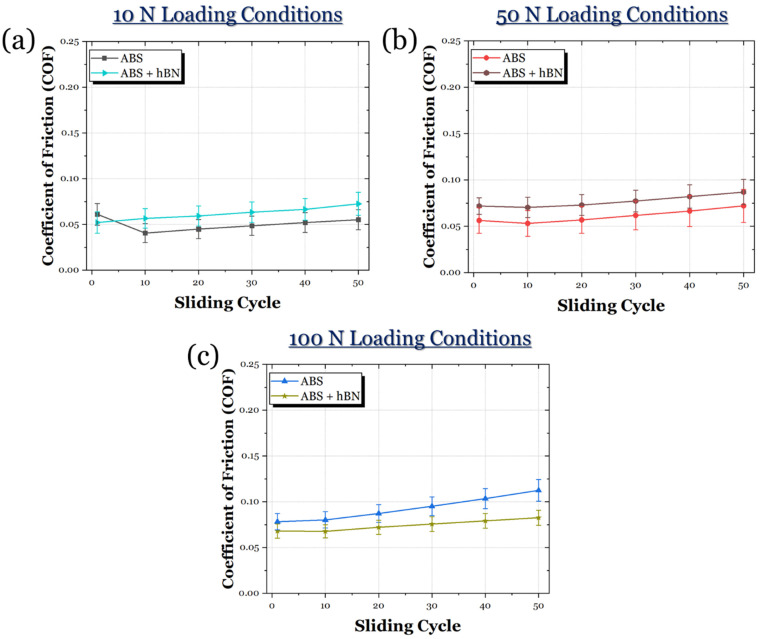
The average COF of the ABS and ABS + hBN specimens in (**a**) 10 N, (**b**) 50 N, and (**c**) 100 N loading conditions.

The frictional curves of the ABS and ABS + hBN composites across 10 N, 50 N, and 100 N loads are shown in Figure 19. For the 10 N loading conditions (Figure 19a), the ABS + hBN specimen appeared to have a fairly stable frictional response. However, relative to the ABS specimen, the ASB + hBN specimen had a slightly greater COF. At the 50 N loading conditions (Figure 19b), the ABS + hBN specimen exhibited the same behavior as the 10 N loading conditions. Specifically, the COF was stable and slightly greater than the ABS specimen. In the 100 N loading conditions (Figure 19c), the COF of the ABS + hBN specimen was lower than the ABS specimen. It could be seen that with this decrease, the frictional curves were also relatively more stable, remaining below 0.1 throughout the entire sliding process.

**Figure 20:**
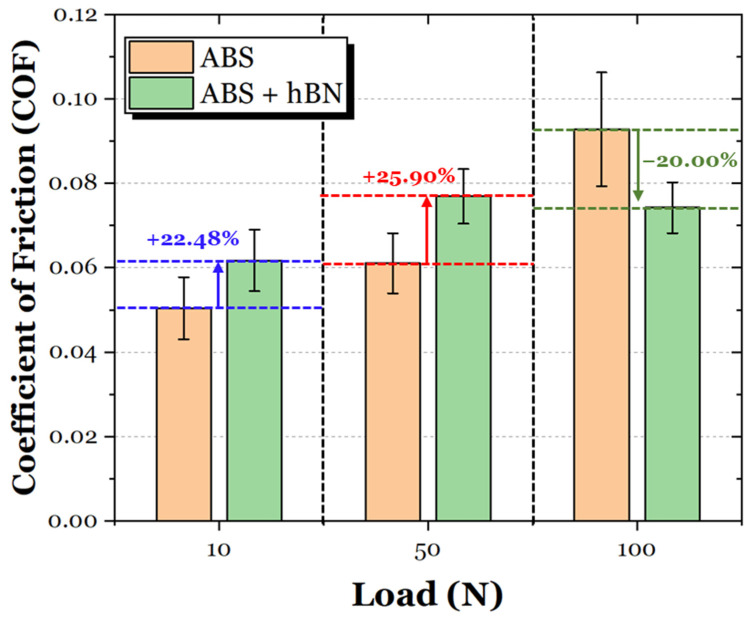
The average COF of the ABS and ABS + hBN specimens in 10 N, 50 N, and 100 N loading conditions.

The average coefficient of friction (COF) values of the ABS and ABS + hBN specimens are shown in Figure 20. At the 10 N loading condition, the COF of the ABS + hBN specimen increased by 22.48%. The 50 N loading condition also had a net increase of 25.90%. However, at the 100 N load, the COF decreased by 20.00%. This trend is somewhat similar to the ABS + Al_2_O_3_ specimen in the sense that the decrease in COF was only prevalent at the 100 N loading conditions.

**Figure 21:**
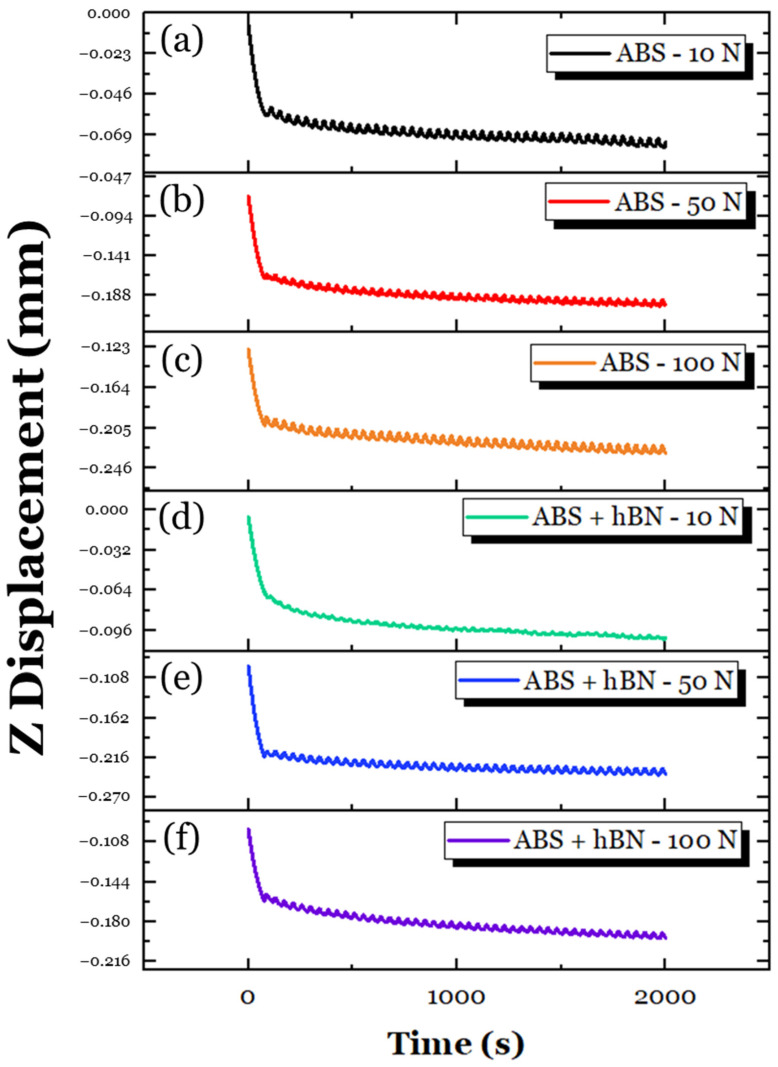
The variation in Z-displacement for the ABS specimen at (**a**) 10 N, (**b**) 50 N, and (**c**) 100 N loading conditions; the variation in Z-displacement for the ABS + hBN specimen at (**d**) 10 N, (**e**) 50 N, and (**f**) 100 N loading conditions.

To better understand the tribological interactions that took place, the change in Z-displacement was also assessed, as shown in Figure 21. Across all loads, it is interesting to note that the Z-displacement of the ABS + hBN specimen was largely stable, unlike the ABS + Al_2_O_3_ specimen. The lack of deviations could be attributed to the lamellar features of the hBN particles. During tribological loading, the weak van der Waals forces of the covalently bonded layers were easily sheared, which produced a lubricating-like effect [53,69]. However, using this logic, the COF for the 10 N and 50 N loads should decrease, similar to the 100 N load. Although the friction should decrease, the lower elastic modulus and higher micropores of the ABS + hBN specimen suggest that greater deformation takes place during sliding. With greater deformation taking place, the contact area of the steel ball also increases, as indicated by the magnitude of change in the Z-depth, which increases friction. Although potentially delaminated hBN particles can assist with friction reduction, the greater contact area likely had a more dominant role in the frictional response. Unlike the 10 N and 50 N loads, the delaminated hBN particles at the 100 N load were likely embedded back into the wear track during sliding. As a consequence, the lubricating features of the hBN particles became more prominent throughout the sliding process.

**Figure 22:**
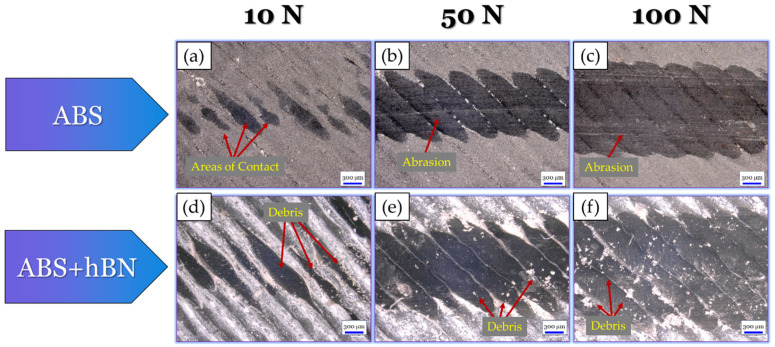
Optical micrographs of the (**a**–**c**) ABS and (**d**–**f**) ABS + hBN wear tracks after tribo-sliding.

Similar to the ABS + Al_2_O_3_ wear track analysis (in Section 3.3.1), optical micrographs of the ABS + hBN wear tracks were taken to better understand the tribological mechanisms that took place. These findings, as well as the micrographs of the ABS wear track, are shown in Figure 22. For reference, although the ABS wear mechanisms are discussed in Section 3.3.1, they are also included in this analysis (in Figure 22a–c) to provide insight into the change in tribological mechanisms from incorporating hBN. Nonetheless, for the ABS + hBN specimen at the 10 N load (Figure 22d), the surface contours are slightly thinned due to the sliding. However, they appear fairly uniform in features. Upon close inspection, evidence of small debris/particles can be seen surrounding the contour lines. This finding suggests that hBN was present within the wear track during sliding, which provided a lubricating-type effect. At the 50 N load (Figure 22e), a greater amount of wear debris was seen. At the 100 N load, wear debris was scattered throughout the entirety of the wear track.

#### 3.3.3. ABS-Al_2_O_3_-hNB Composites

**Figure 23:**
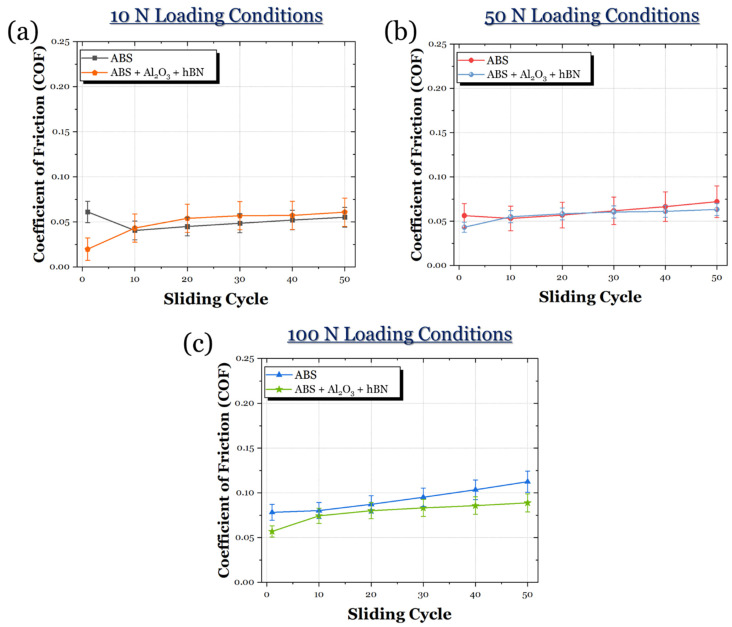
The average COF of the ABS and ABS + Al_2_O_3_ + hBN specimens in (**a**) 10 N, (**b**) 50 N, and (**c**) 100 N loading conditions.

The frictional curves of the ABS and ABS + Al_2_O_3_ + hBN specimens in 10 N, 50 N, and 100 N loading conditions are shown in Figure 23. At the 10 N loading condition (Figure 23a), the ABS + Al_2_O_3_ + hBN specimen exhibited a frictional response that was somewhat similar to the ABS specimen. However, the COF of the ABS + Al_2_O_3_ + hBN specimen appeared to be slightly greater than the ABS specimen. At the 50 N loading condition (Figure 23b), the ABS + Al_2_O_3_ + hBN specimen experienced a frictional response that was also similar to the ABS specimen. However, the COF appeared to be slightly reduced. At the 100 N load (Figure 23c), the ABS + Al_2_O_3_ + hBN specimen exhibited a decreased COF again compared to the ABS specimen. Compared to the 50 N loading condition, there was a greater difference in COF compared to the ABS specimen.

**Figure 24:**
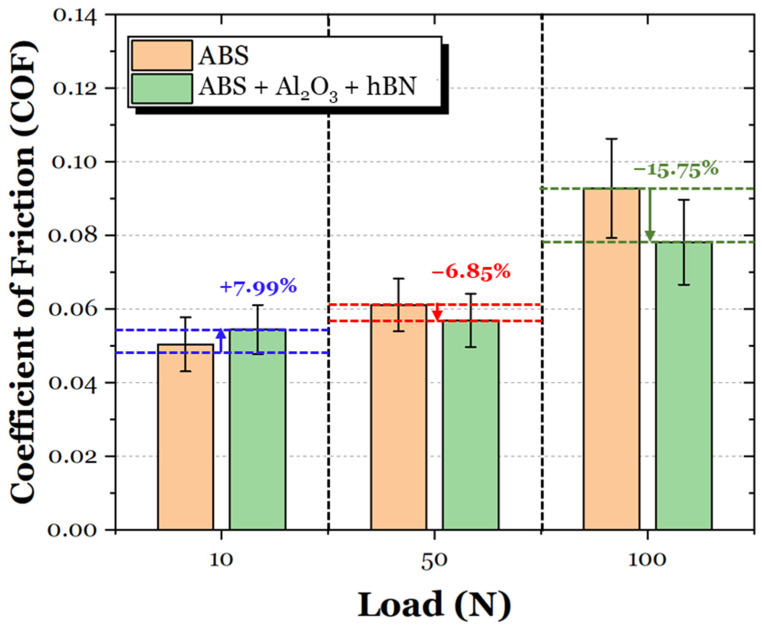
The average COF of the ABS and ABS + Al_2_O_3_ + hBN specimens in 10 N, 50 N, and 100 N loading conditions.

The average frictional response of the ABS and ABS + Al_2_O_3_ + hBN specimens is shown in Figure 24. At the 10 N load, the COF of the ABS + Al_2_O_3_ + hBN specimen was 7.99% greater than the ABS specimen. Although greater, the obtained COF was less than ABS + Al_2_O_3_ and ABS + hBN specimens at the same load. Interestingly enough, at the 50 N load, the COF of the ABS + Al_2_O_3_ + hBN specimen decreased by 6.85%. At the 100 N load, the COF again decreased by 15.75%, indicating that the combination of Al_2_O_3_ and hBN had a positive effect on friction resistance.

**Figure 25:**
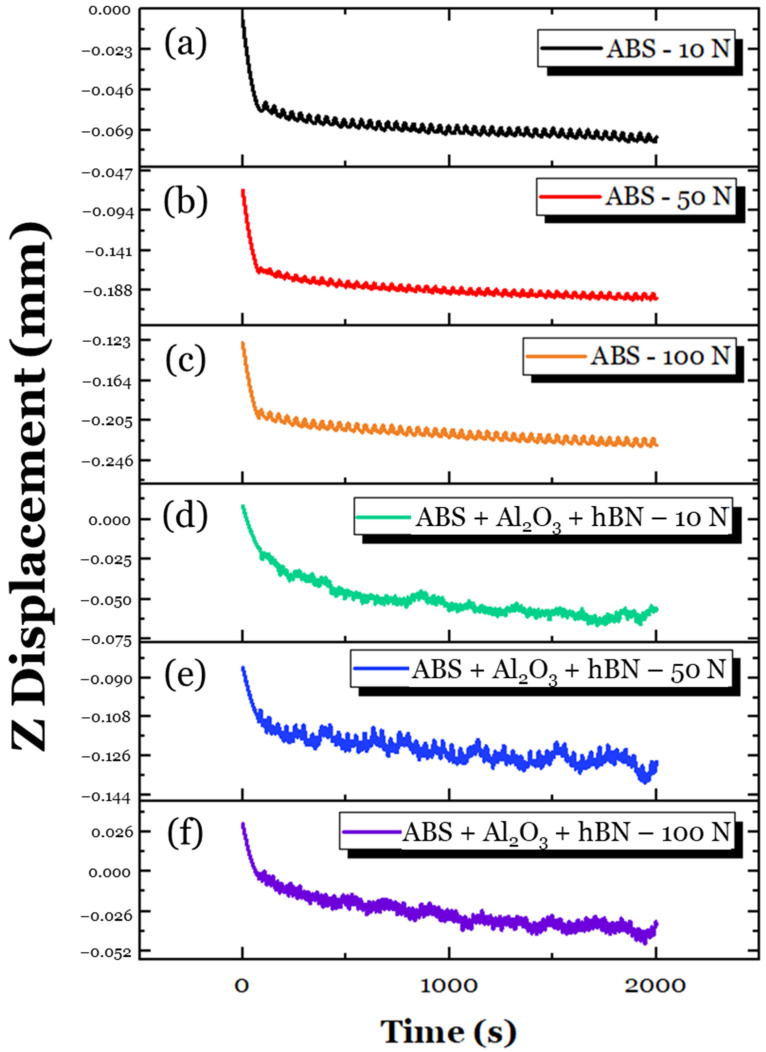
The variation in Z-displacement for the ABS specimen at (**a**) 10 N, (**b**) 50 N, and (**c**) 100 N loading conditions; the variation in Z-displacement for the ABS + Al_2_O_3_ + hBN specimen at (**d**) 10 N, (**e**) 50 N, and (**f**) 100 N loading conditions.

Using the same approach as the ABS + Al_2_O_3_ and ABS + hBN tribological analysis, the change in Z-displacement of the ABS + Al_2_O_3_ + hBN specimen was studied, as shown in Figure 25. Across all loads, there appeared to be a few fluctuations in the Z displacement, suggesting that potential particle delamination/third-body interactions took place. It is conspicuous to note that the fluctuations were more intense than the ABS + hBN Z–displacement curves but less intense than the ABS + Al_2_O_3_ Z–displacement curves. This finding suggests that both the Al_2_O_3_ and hBN particles have simultaneous interactions during sliding. In relation to the COF, it is likely that at the 10 N load, the potential delamination of particles could have abrasive effects during sliding [70]. This is because the load is relatively light, which reduces the likelihood of the particles being adhesively transferred back to the wear track. At the 50 N and 100 N loads, the Al_2_O_3_ particles likely helped with the load distribution, whereas the hBN particles provided a lubricating-like effect. This could be due to greater loading, as there is a greater chance of the reinforcement particles being re-embedded into the wear track. Although the elastic modulus of the ABS + Al_2_O_3_ + hBN specimen is less than the ABS specimen (which could result in greater deformation, as indicated by the fluctuations of Z-displacement), the influence of Al_2_O_3_ and hBN composites had a positive effect on friction resistance.

**Figure 26:**
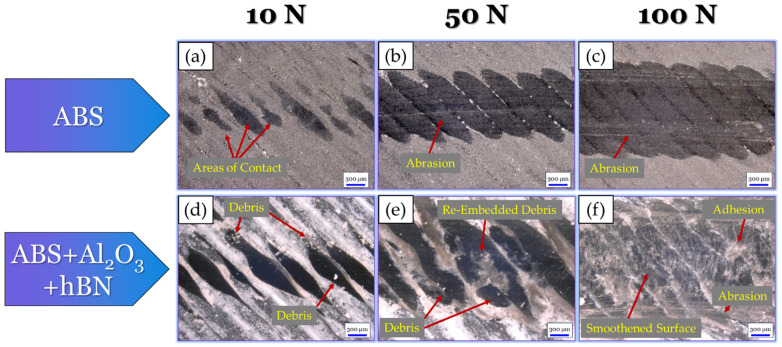
Optical micrographs of the (**a**–**c**) ABS and (**d**–**f**) ABS + Al_2_O_3_ + hBN wear tracks after tribo-sliding.

Optical micrographs of ABS + Al_2_O_3_ + hBN and ABS (which is used as a reference) wear tracks are shown in Figure 26. As previously mentioned in Section 3.3.2, the analysis of the ABS wear track (Figure 26a–c) is discussed in Section 3.3.1. For the ABS + Al_2_O_3_ + hBN specimen at the 10 N load (Figure 26d), similar wear track features as the ABS + hBN specimen (Figure 22d) can be seen. As the load increases to 50 N (Figure 26e), the wear track increases in size. In addition, evidence of debris and adhesively re-embedded debris within the wear track can be seen. At the 100 N load (Figure 26f), the wear track size again increases with similar features to the 50 N wear track. Aside from these features, slight signs of abrasion are also evident near the edges of the wear track.

#### 3.3.4. Comparative Assessment

**Figure 27:**
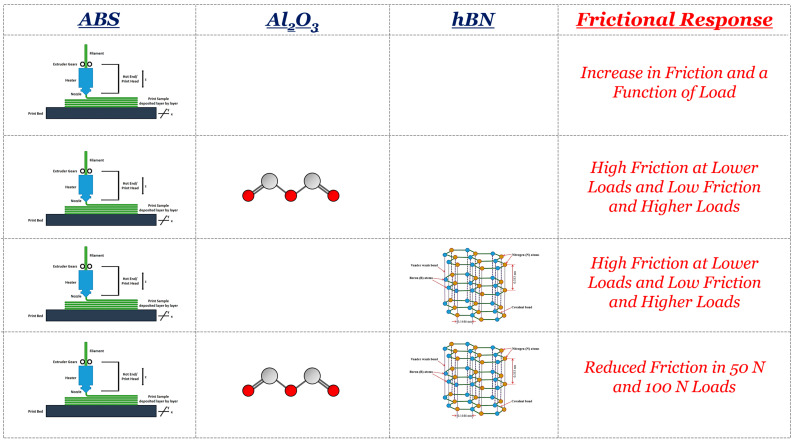
The tribological performance table matrix of 3D-printed ABS, ABS, ABS + Al_2_O_3_, ABS + hBN, and ABS + hBN + Al_2_O_3_.

Based on these findings, it can be seen that the tribological mechanisms of 3D-printed composite-sprayed ABS are quite complicated. To obtain a comprehensive understanding of the effects of hBN and Al_2_O_3_, a performance table explaining the general tribological takeaways was fabricated. This performance table is shown in Figure 27. For the ABS substrate, the tribological mechanisms were relatively straightforward. As the tribo-load increased, the friction also increased due to the increase in the contact area along the surface. However, with the incorporation of Al_2_O_3_, the influence of particle delamination and potential third-body wear increased the friction at lower loads (i.e., 10 N and 50 N). When the load was 100 N, the loosened Al_2_O_3_ particles were likely adhesively bonded back to the surface, which then supported the externally applied load, thus reducing the COF. The same phenomenon also occurred for the hBN particles. However, when they were added, they acted as a solid lubricant and reduced friction. When Al_2_O_3_ and hBN were simultaneously added, they provided load-bearing and solid-lubricating characteristics, which reduce friction at intermediate (i.e., 50 N) and high (i.e., 100 N) loads.

**Figure 28:**
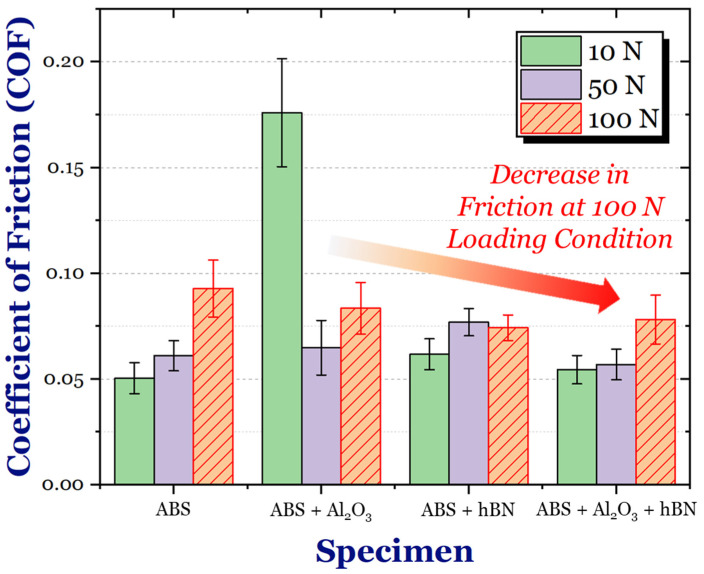
A comparative overview of the frictional response of the ABS, ABS, ABS + Al_2_O_3_, ABS + hBN, and ABS + hBN + Al_2_O_3_ specimens at 10 N, 50 N, and 100 N loads.

To better observe the relationship between reinforcement addition, normal load, and friction, the average COF between Figure 11, Figure 14 and Figure 17 were compiled, as shown in Figure 28. It is clear to see that the addition of Al_2_O_3_ and hBN particles (in their independent and mixed states) had a positive effect on the frictional resistance of 3D-printed ABS in extreme loading conditions. Based on these findings, it can be suggested that the interlayer addition of ceramic and self-lubricating composites for 3D-printed ABS is a viable technique to control friction resistance.

## 4. Conclusions

In this work, the influence of interlayer-sprayed Al_2_O_3_ and hBN on the mechanical and tribological properties of FDM’d ABS was investigated. The key takeaways are as follows:

1. The addition of Al_2_O_3_ and hBN resulted in a large formation of micropores along the ABS structure. This finding was attributed to the interactions of the reinforcement and alcohol during the cooling process, which likely altered the cooling rates of the surrounding filament.
2. In tensile-loading conditions, the presence of Al_2_O_3_ and hBN reinforcements degraded the mechanical properties of the ABS substrate. Between Al_2_O_3_ and hBN, the addition of hBN had the most negative impact on tensile strength. This finding can be likely attributed to the lack of cohesive bonding between the ABS layers due to the interactions of the reinforcement and alcohol during the cooling process. Also, the presence of micropores contributed to this decrease.
3. In sliding conditions, the individual addition of Al_2_O_3_ and hBN improved the frictional resistance at 100 N loading conditions. When added together, the frictional resistance was improved at 50 N and 100 N loading conditions. The decrease in friction was attributed to the ability of the Al_2_O_3_ to support the tribo-load, whereas hBN acted as a solid lubricant due to its lamellar structure.

Although the mechanical properties of the reinforcement-added ABS substrates were degraded, the tribological performance was improved. However, it should be mentioned that the tribological performance was only improved at higher loads. Nonetheless, this work demonstrates that there is viability in adding Al_2_O_3_ and hBN to FDM’d ABS to control its frictional resistance.

## Figures and Tables

**Table 1:** The 3D printing settings used for sample creation.

3D Printing Parameters	
Layer height	0.25 mm
Filament diameter	1.75 ± 0.02 mm
Nozzle temperature	255 °C
Nozzle diameter	0.4 mm
Print bed temperature	100 °C
Infill	100%
Top/bottom infill pattern	Rectilinear
Internal infill pattern	Grid

**Table 2:** The list of composite sample sets fabricated in this work.

Specimen Name	Reinforcement	Reinforcement Quantity
ABS	None	No material added
ABS + Al_2_O_3_	Al_2_O_3_	2 g Alumina per 130 mL isopropyl alcohol
ABS + hBN	hBN	2 g hBN per 130 mL isopropyl alcohol
ABS + Al_2_O_3_ + hBN	Al_2_O_3_ and hBN	1 g Alumina, 1 g hBN per 130 mL isopropyl alcohol

## Data Availability

Data is contained within the article.
